# Supplementary material for: Structural analysis of the overoxidized Cu/Zn-superoxide dismutase in ROS-induced ALS filament formation
Source: Commun Biol. 2022 Oct 12;5:1085. doi: 10.1038/s42003-022-04017-0 (PMC9556535; doi:10.1038/s42003-022-04017-0)
Supplement: Supplementary file 2 — Supplementary Information [file 42003_2022_4017_MOESM2_ESM.pdf]

Structural analysis of the overoxidized Cu/Zn-superoxide dismutase in ROS-induced ALS filament formation

Yeongjin Baek[1], Tae-Gyun Woo[3], Jinsook Ahn[1,2], Dukwon Lee[1], Yonghoon Kwon[1], Bum-Joon Park[3], and Nam-Chul Ha[1,2,*]

[1]Department of Agricultural Biotechnology, and Research Institute of Agriculture and Life Sciences, CALS, Seoul National University, Seoul 08826, Republic of Korea

[2]Center for Food and Bioconvergence, Seoul National University, Seoul 08826, Republic of Korea

[3]Department of Molecular Biology, College of Natural Science, Pusan National University, Busan 46241, Republic of Korea

*To whom correspondence should be addressed NCH (e-mail: hanc210@snu.ac.kr, Tel.: +82-2-880-4853, Fax: +82-2-873-5095, ORCID ID: 0000-0003-4813-748X)

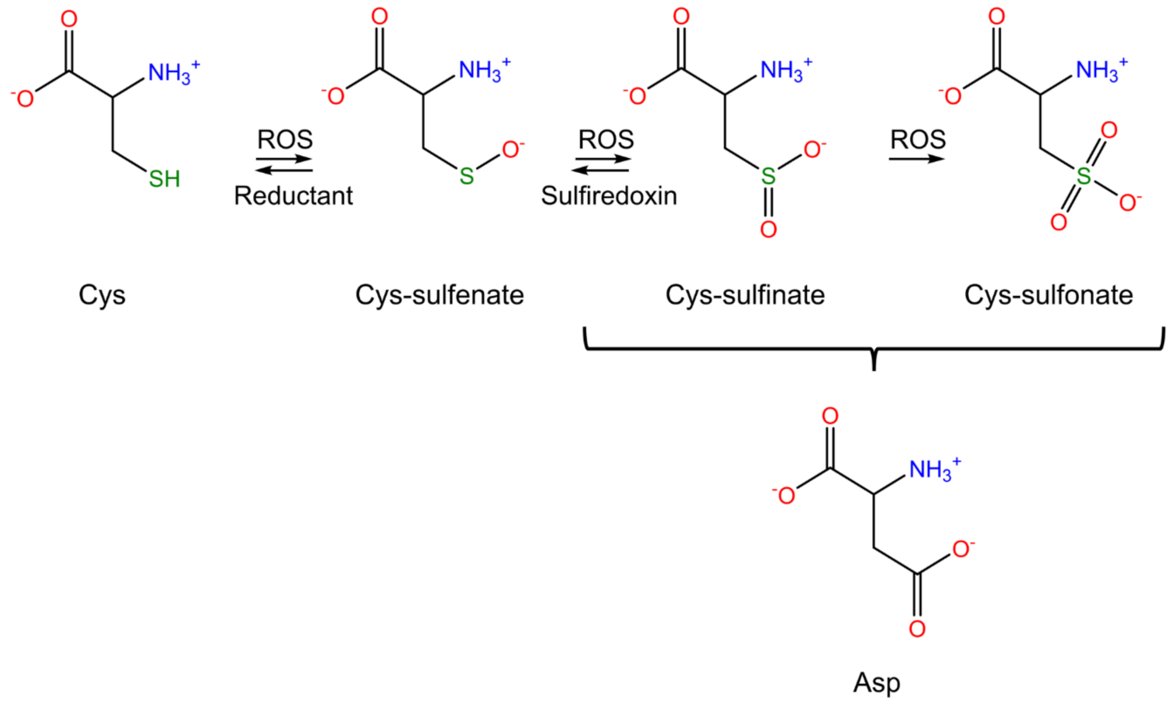

Supplementary Figure 1. Schematic diagram of cysteine oxidation and aspartate to mimic overoxidation of cysteine.

Cysteine is oxidized into cysteine-sulfenate in the presence of ROS stress. Cysteine-sulfenate is further oxidized into cysteine-sulfinate and then cysteine-sulfonate by the ROS stress. Cysteine-sulfenate can be reduced back to cysteine in the presence of the reducing agent. Cys-sulfinate can be reduced by specific enzymes, such as the chaperone sulfiredoxin requiring ATP. Cysteine-sulfonate is not known to be reduced under normal conditions. Aspartic acid mimics cysteine-sulfinate and cysteine-sulfonate by the charge and atomic arrangement.

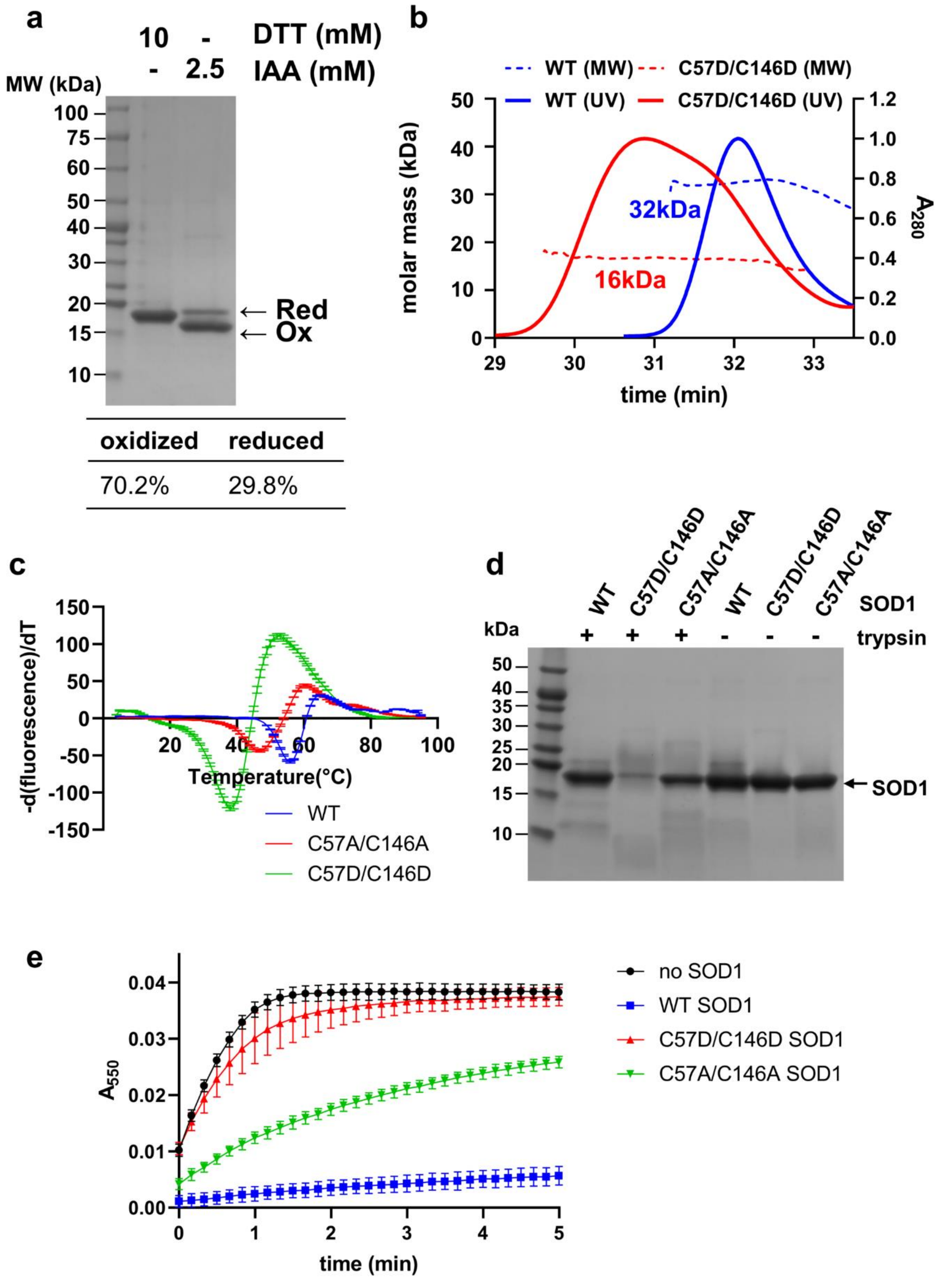

Supplementary Figure 2. Supporting evidence for Fig. 1

a. Redox state of the purified wild-type SOD1 protein. The wild-type protein solution at a concentration of 1 mg/ml was incubated with 10 mM DTT or 2.5 mM iodoacetamide (IAA) for 30 min at 37°C and was assessed by SDS-PAGE. The redox states of SOD1 proteins are shown on the right of the SDS polyacrylamide gel image. All the cysteines in SOD1 are reduced (red), or the intramolecular disulfide bond is formed between Cys57 and Cys146 (Ox). The reduced or oxidized portion of the wild-type SOD1 protein was quantitatively analyzed using ImageJ[1] and represented as a ratio to total protein concentration at the below of the SDS-PAGE image.

b. SEC-MALS profiles of the purified wild-type (blue) and C57D/C146D mutant (red) SOD1 proteins in the presence of 5 mM DTT. The UV absorbance at 280 nm (A_{280} at the right Y-axis) of the SEC is represented by solid lines. The molecular mass (the left Y-axis) based on MALS is represented by a dotted line. The average molar mass of wild-type or C57D/C146D mutant SOD1 is indicated under the dotted lines.

c. Tm measurement by the thermal shift assay of the wild-type SOD1 protein (WT, blue line), C57A/C146A mutant SOD1 protein (green line), and C57D/C146D mutant SOD1 protein (red line). The data points are the first derivate of the denaturation curve and are displayed by the average of three replicates with ±SD.

d. Proteolytic digestion of the wild-type SOD1, C57A/C146A mutant SOD1, and C57D/C146D mutant SOD1 proteins by trypsin. The proteins were digested by trypsin at 37ºC for 30 min and subjected to SDS-PAGE in the presence of 10 mM DTT. The SOD1. The band of the SOD1 protein is indicated by an arrow to the right of the SDS polyacrylamide gel image.

e. Superoxide dismutase activities of SOD1 proteins. Absorbance at 550 nm from the reduction of the cytochrome c indicates the amount of superoxide ion produced by *E. coli* C43A/C48A mutant RclA protein in the presence of NADH[2]. The added wild-type or the mutant SOD1 protein diminishes the superoxide production rates. The SOD1-free reaction gave a linear increase in cytochrome *c* reduction for 1 min (black), and most of the superoxide anion accumulation by RclA was scavenged by wild-type SOD1 protein (blue). In contrast, the C57D/C146D mutant SOD1 protein almost failed to scavenge

superoxide anions (red). The superoxide scavenging ability of C57A/C146A mutant SOD1 (green) was better than that of C57D/C146D mutant SOD1 and less than that of wild-type SOD1. The graph shows the mean ± SD from six individual experiments.

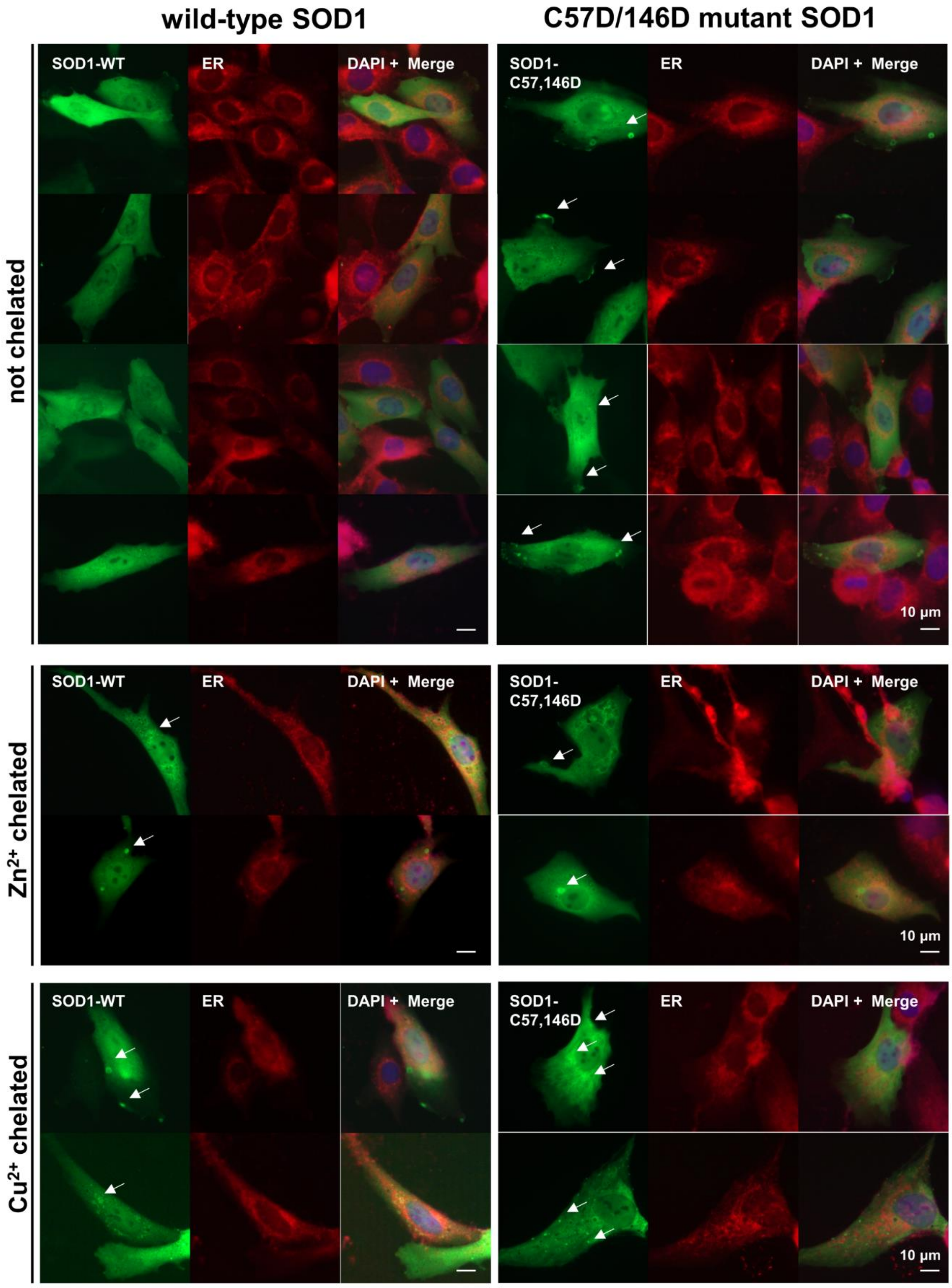

Supplementary Figure 3. Immunofluorescence staining of overexpressed SOD1 in SK-N-SH cells (More images from Fig. 2B).

SK-N-SH cells transfected with pcDNA3.1 expressing the wild-type SOD1 (left) or the C57D/C146D SOD1 (right) gene were incubated with Zn^{2+} chelator (TPEN, middle panels) or Cu^{2+} chelator (ATN-224, lower panels) or without any treatment (upper panels) for 12 hr. Cells were visualized by the appropriate antibody and dyes: SOD1 in green, ER in red, and DNA in blue. Colocalization of SOD1 with ER shows an orange field. Arrows indicate the cytoplasmic accumulation of SOD1 proteins. White arrows indicated cells with SOD1 inclusions. Scale bar; 10 μm.

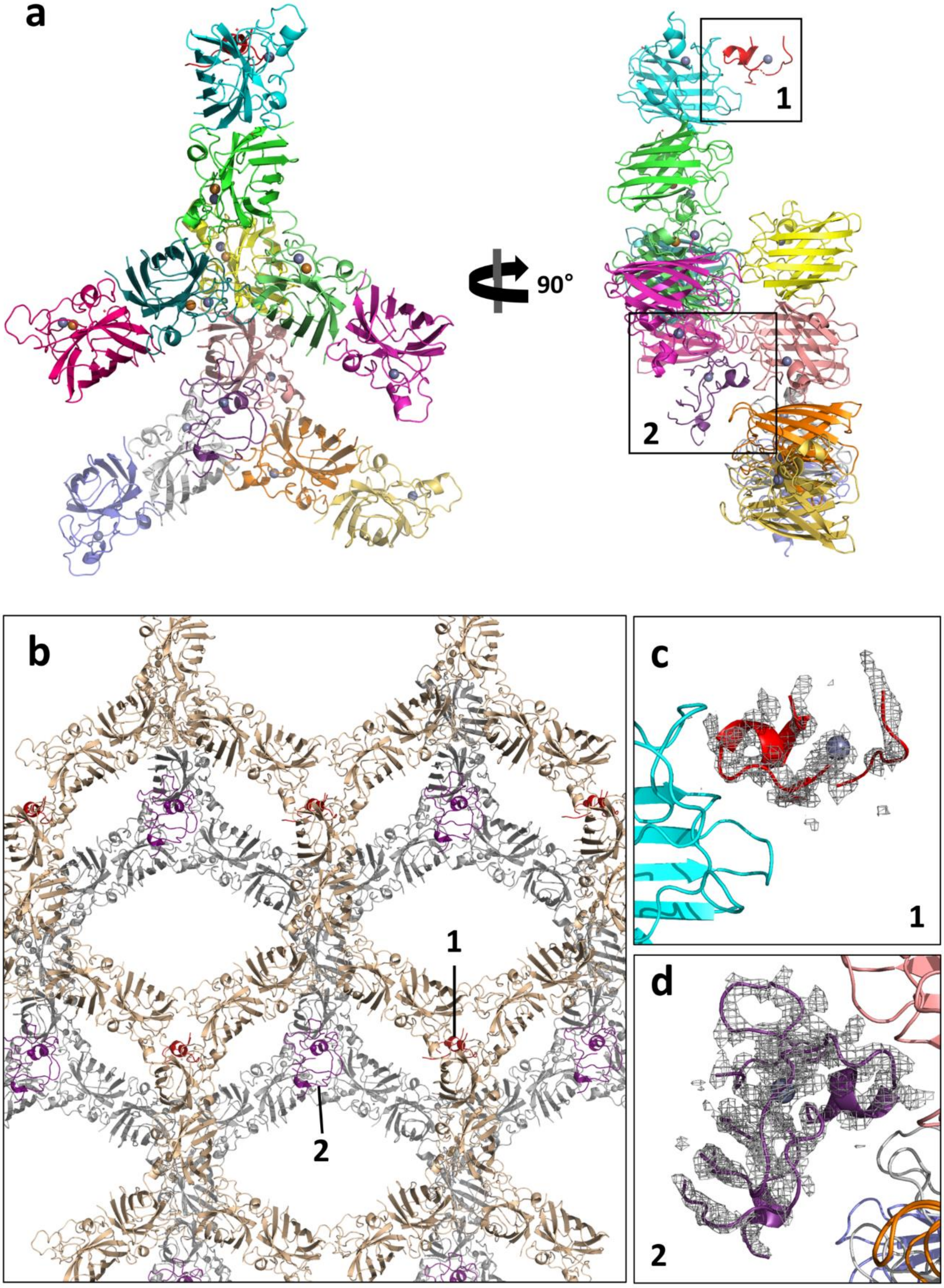

Supplementary Figure 4. Crystal structure of the wild-type SOD1.

a. Structure of the asymmetric unit of the wild-type SOD1 in the ribbon representations. The asymmetric

unit consists of twelve complete models (cyan, green, pale green, forest, magenta, hot pink, yellow, light pink, gray, orange, pale yellow, and light blue) and two partial models (purple, red). The gold spheres represent Cu^{2+}, and the gray spheres represent Zn^{2+}. Two partial models are marked as two small boxes. Box 1 indicates partial model 1, and box 2 indicates partial model 2.

b. Molecular arrangement of the wild-type SOD1 in the crystal. The complete and partial models are arranged in two layers (upper layer in wheat, lower layer in gray) in the ribbon representations. The partial models were in the space between the honeycomb: partial model 1 in red and partial model 2 in purple.

c. Partial model 1 of the wild-type SOD1 enlarged from box 1 of Fig S3A in the ribbon diagram (red). The 2Fo-Fc electron density map for partial model 1 is contoured at 1.5 σ (gray).

d. Partial model 2 of the wild-type SOD1 enlarged from box 2 of Fig S3A in the ribbon diagram (purple). The 2Fo-Fc electron density map of partial model 2 is contoured at 1.5 σ (gray).

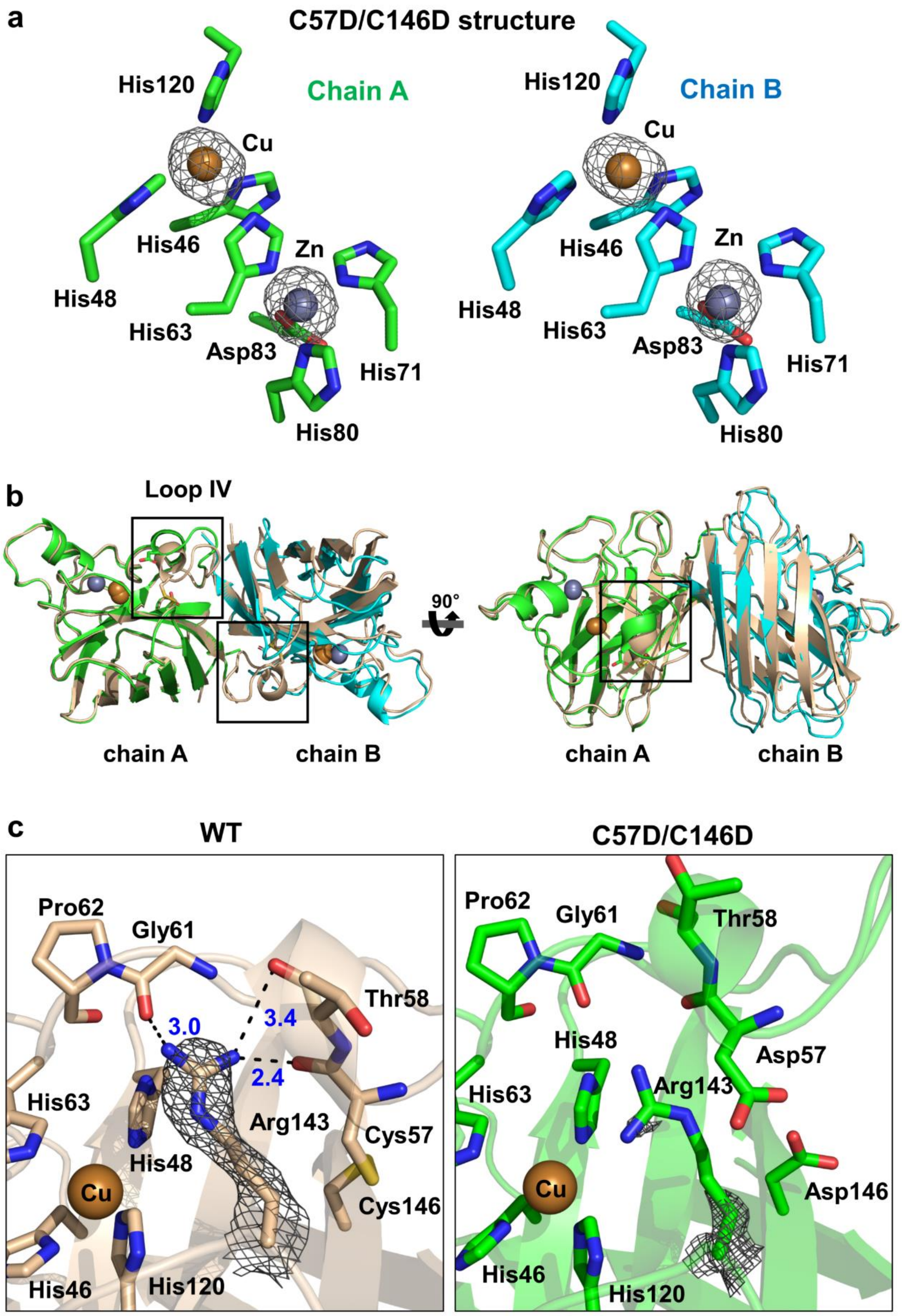

Supplementary Figure 5. Structural comparison of the C57D/C146D mutant with wild-type SOD1.

a. The omit map for copper ion (gold sphere) and zinc ion (silver sphere) from the C57D/C146D mutant

SOD1. The |(Fo)-(Fc)| difference density is contoured at 3 σ (gray). Histidine residues interacting with bound metal ions are labeled and indicated in stick representations.

b. Dimer structure of C57DC146D mutant SOD1 aligned with the wild-type (WT) SOD1 dimer structure. The C57D/C146D mutant SOD1 is colored cyan (chain A) and green (chain B), and the wild-type SOD1 is colored wheat. The ribbon representation depicts each subunit. The gold spheres represent Cu^{2+}, and the gray spheres represent Zn^{2+} ions. The black square box indicates loop IV of wild type and C57D/C146D mutant SOD1.

c. 2Fo-Fc Electron density map of the Arg143 side chain in the wild-type SOD1 (left, wheat) and C57D/C146D mutant SOD1 structure (right, green). Each residue is labeled and indicated in the stick representations. The gold spheres represent Cu^{2+}. Distances are in Å (blue). The 2Fo-Fc electron density map for the Arg144 residue is contoured at 1.5 σ (gray).

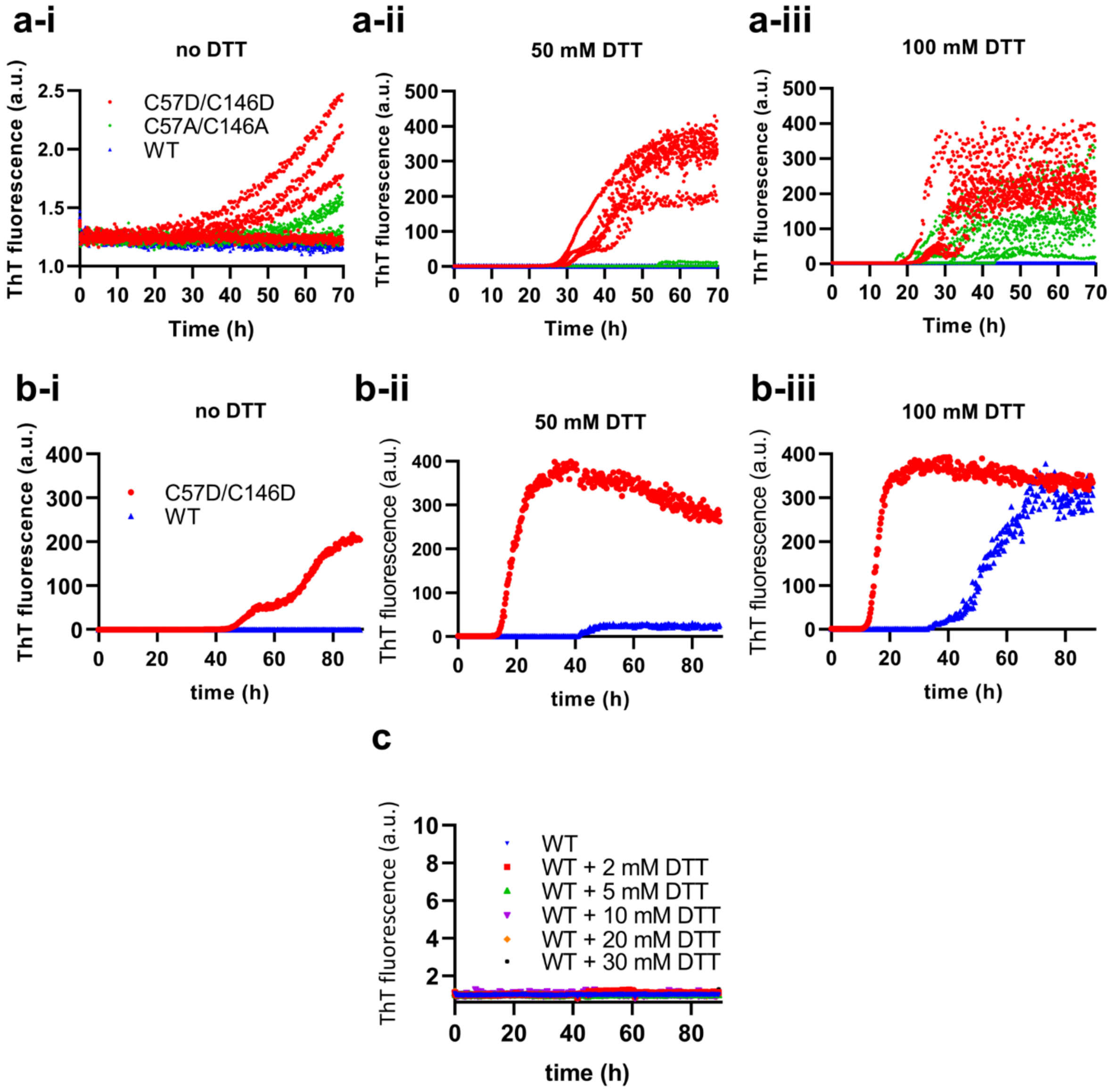

Supplementary Figure 6. Thioflavin T (ThT) assay used to measure the amyloid-like filament formation of the C57D/C146D mutant SOD1 protein.

a. Thioflavin T (ThT) fluorescence intensities representing the filament formation of the C57D/C146D mutant SOD1 (red), C57A/C146A mutant SOD1 (green), and wild-type SOD1 (WT, blue) proteins in the reaction buffer containing 0 mM DTT (A-i), 50 mM DTT (A-ii), and 100 mM DTT (A-iii). The data points are represented by the mean values of six independent experiments. Individual raw datasets of the three independent experiments are presented in the same color.

b. Thioflavin T (ThT) fluorescence intensities representing filament formation of the C57D/C146D mutant SOD1 (red) and the wild-type SOD1 (WT, blue) proteins in reaction buffer containing 0 mM DTT (A-i), 50 mM DTT (A-ii), and 100 mM DTT (A-iii). The data points are represented by the mean values of three independent experiments.

c. Thioflavin T (ThT) fluorescence intensities representing filament formation of the wild-type SOD1 proteins incubated with 0 mM (black), 5 mM (blue), 10 mM (purple), 20 mM (red) and 30 mM (green) DTT. The data points are represented by the mean values of three independent experiments.

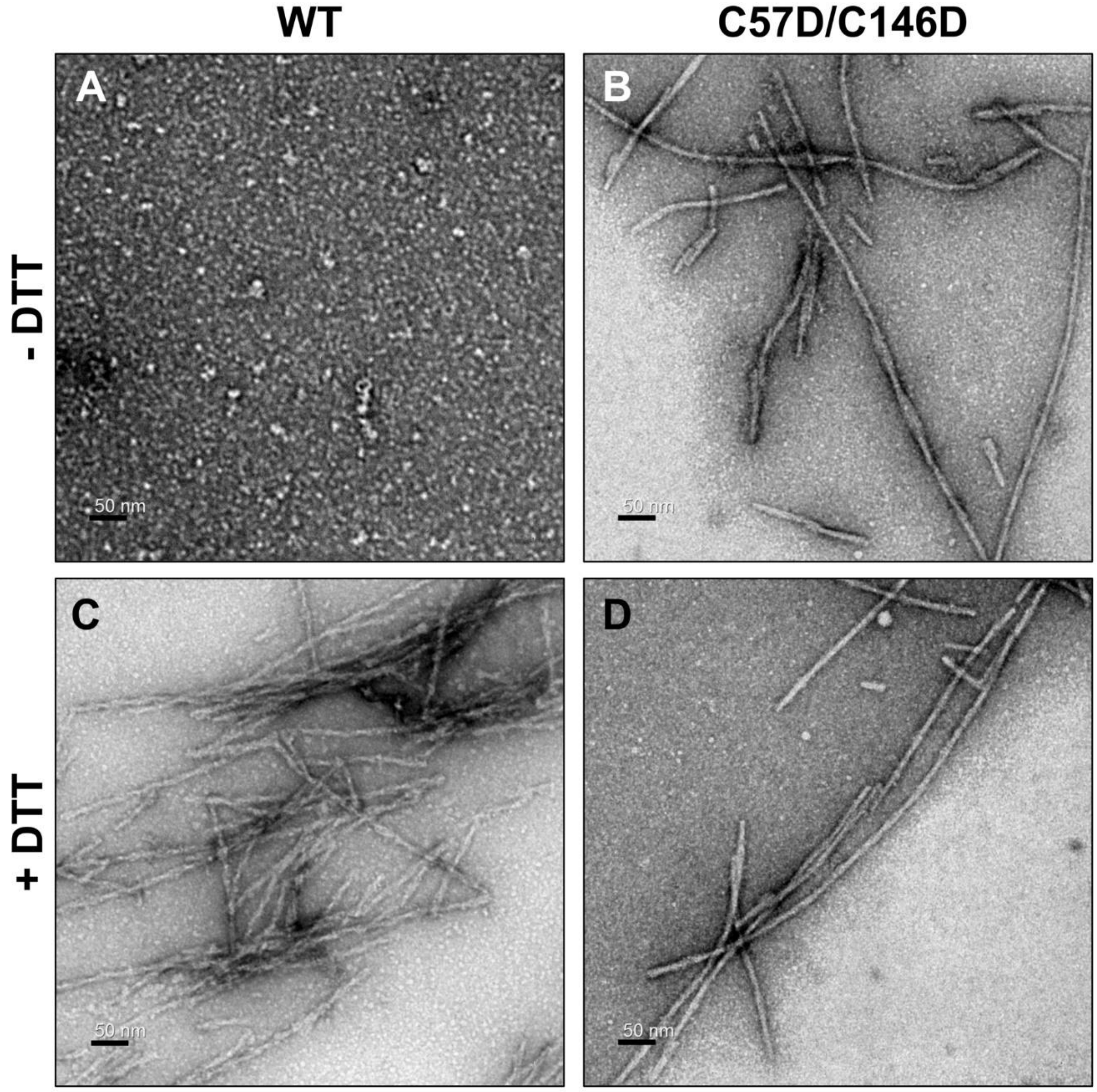

Supplementary Figure 7. Morphology of the C57D/C146D mutant SOD1 and wild-type SOD1 protein aggregates.

Negative-stain transmission electron micrographs of the wild-type and C57D/C146D mutant SOD1 protein aggregates. The SOD1 protein solution was incubated for 90 h in the presence or absence of 50 mM DTT. The C57D/C146D mutant or wild-type SOD1 protein was incubated in the absence of DTT (**a**, **b**, respectively) or in the presence of 50 mM DTT (**c**, **d**, respectively). Scale bars indicate 50 nm.

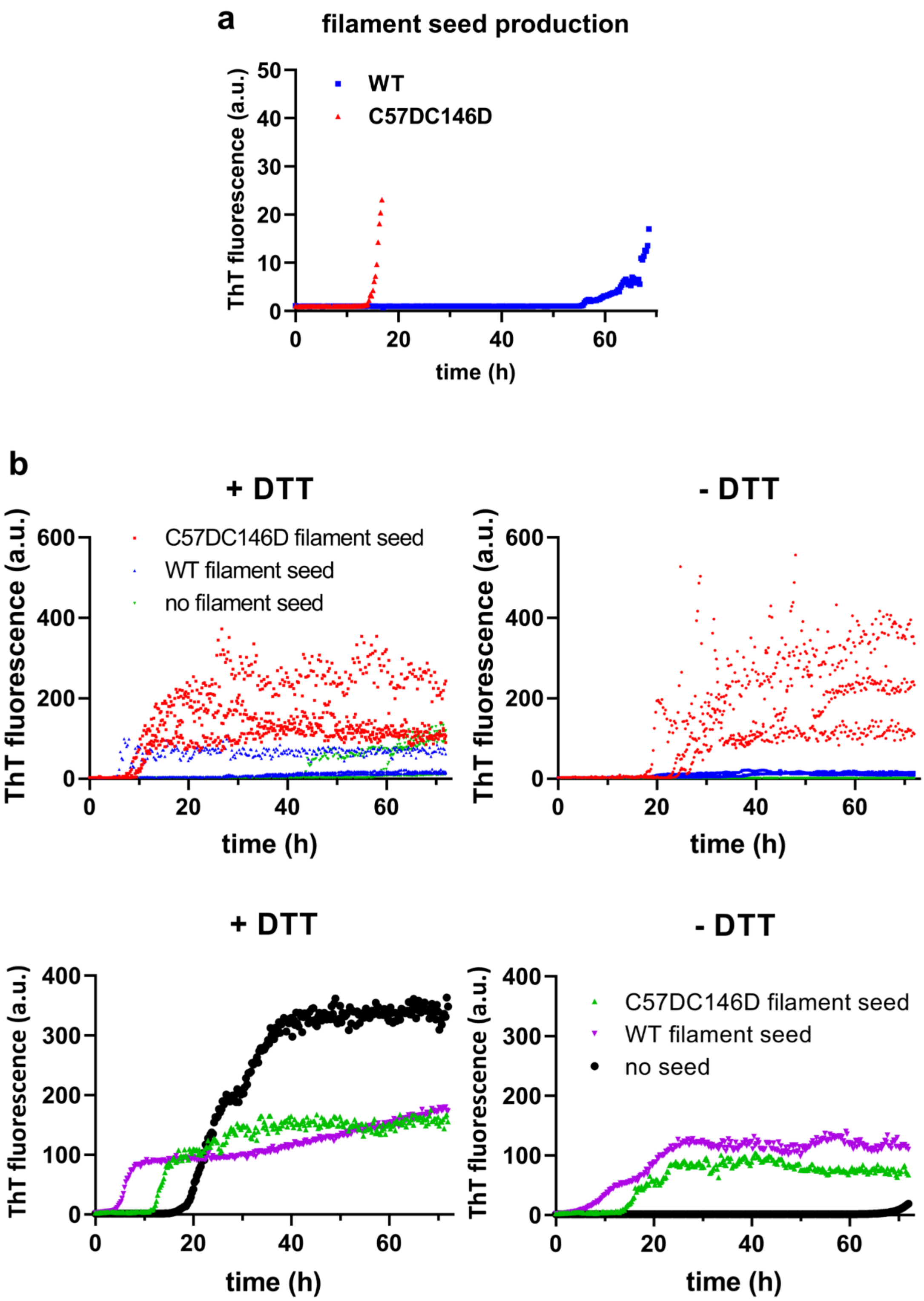

Supplementary Figure 8. Seeding activity of the wild-type or C57D/C146D mutant SOD1-driven filament seeds.

a. Production of wild-type or C57D/C146D mutant SOD1 filament seeds. The wild-type SOD1 (orange)

or C57D/C146D mutant SOD1 (blue) protein solution was incubated until the thioflavin T (ThT) fluorescence reached approximately 20, and the degree of fluorescence indicated the initial stage of exponential growth of the SOD1 protein filament.

b. ThT fluorescence intensities of the wild-type SOD1 protein solution in the preformed wild-type or C57D/C146D mutant SOD1 filament in the absence of DTT (left) or the presence of 50 mM DTT (right). Red, wild-type SOD1 protein incubated with C57D/C146D SOD1 filament seeds. Blue, wild-type SOD1 protein incubated with wild-type SOD1 filament seeds. Green, wild-type protein incubated in the absence of preformed filament seeds. Individual raw datasets of the three independent experiments are presented in the same color.

c. ThT fluorescence intensities of C57D/C146D mutant SOD1 protein solution incubated with preformed wild-type or C57D/C146D mutant SOD1 filament seeds in the absence of DTT (left) or the presence of 50 mM DTT (right). Green, C57D/C146D mutant SOD1 protein incubated with C57D/C146D filament seeds. Purple, C57D/C146D mutant SOD1 protein incubated with wild-type filaments. Black, C57DC146D mutant SOD1 protein incubated without preformed filament seeds. The intensities are shown as the mean of three independent experiments.

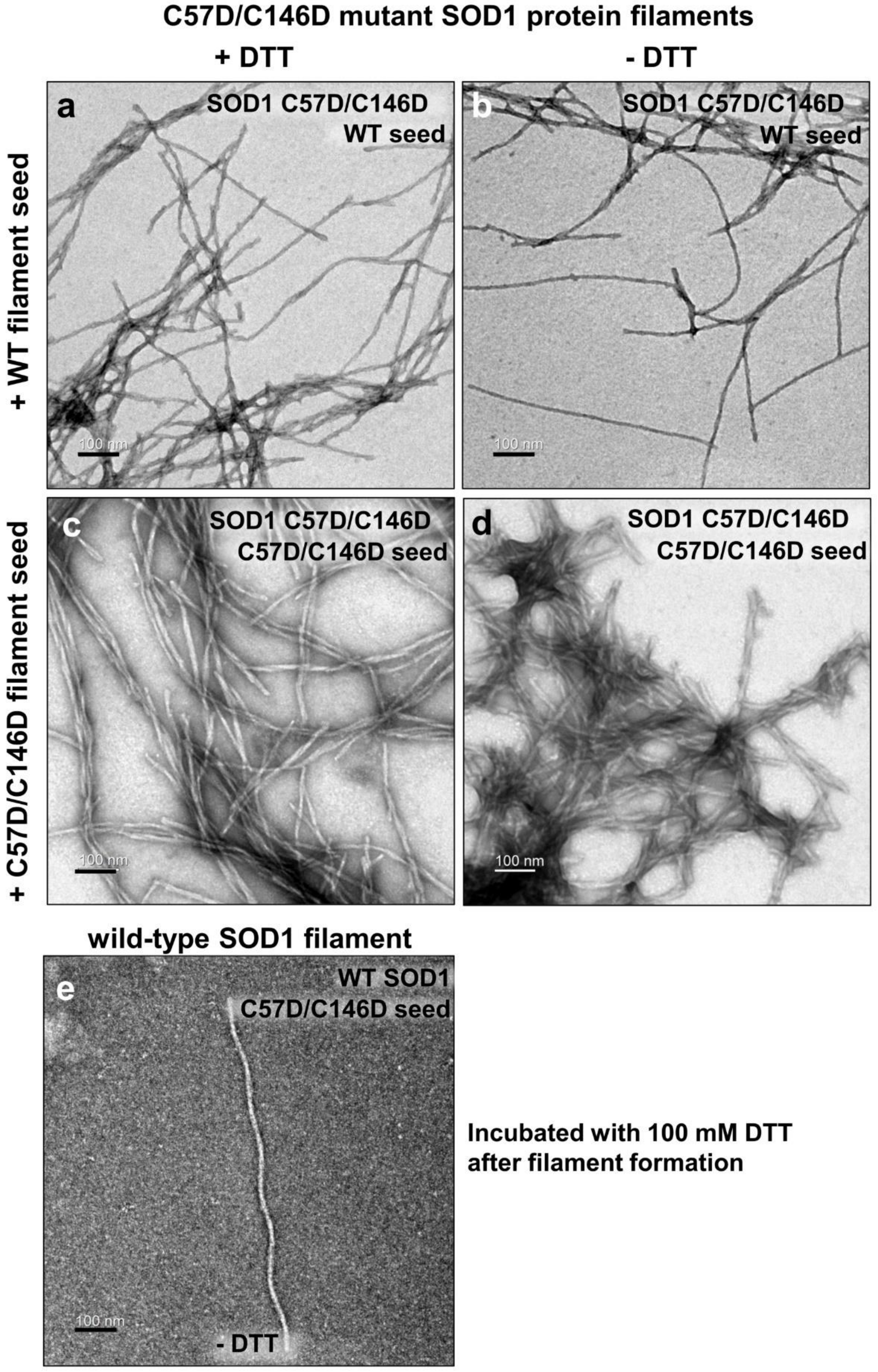

Supplementary Figure 9. Morphology of the C57D/C146D mutant or wild-type SOD1 protein filaments formed with wild-type or C57D/C146D filament seeds.

a-d. Negative-stain transmission electron micrographs of C57D/C146D mutant SOD1 proteins incubated with seeds for 90 h in the presence or absence of 50 mM DTT. The C57D/C146D mutant SOD1 protein solution was incubated with wild-type filament seeds in the absence of DTT (**a**) or the presence of DTT (**b**). The C57D/C146D mutant SOD1 protein solution was incubated with C57D/C146D filament seeds in the absence of DTT (**c**) or the presence of DTT (**d**). Scale bars indicate 100 nm.

e. Negative-stain transmission electron micrograph of the wild-type SOD1 protein filaments formed with C57DC146D mutant filaments as seeds in the absence of DTT. After filament formation, the wild-type SOD1 protein filaments were further incubated with 100 mM DTT for 30 min at 37°C with continuous shaking before negative-stain sampling. Scale bars indicate 100 nm.

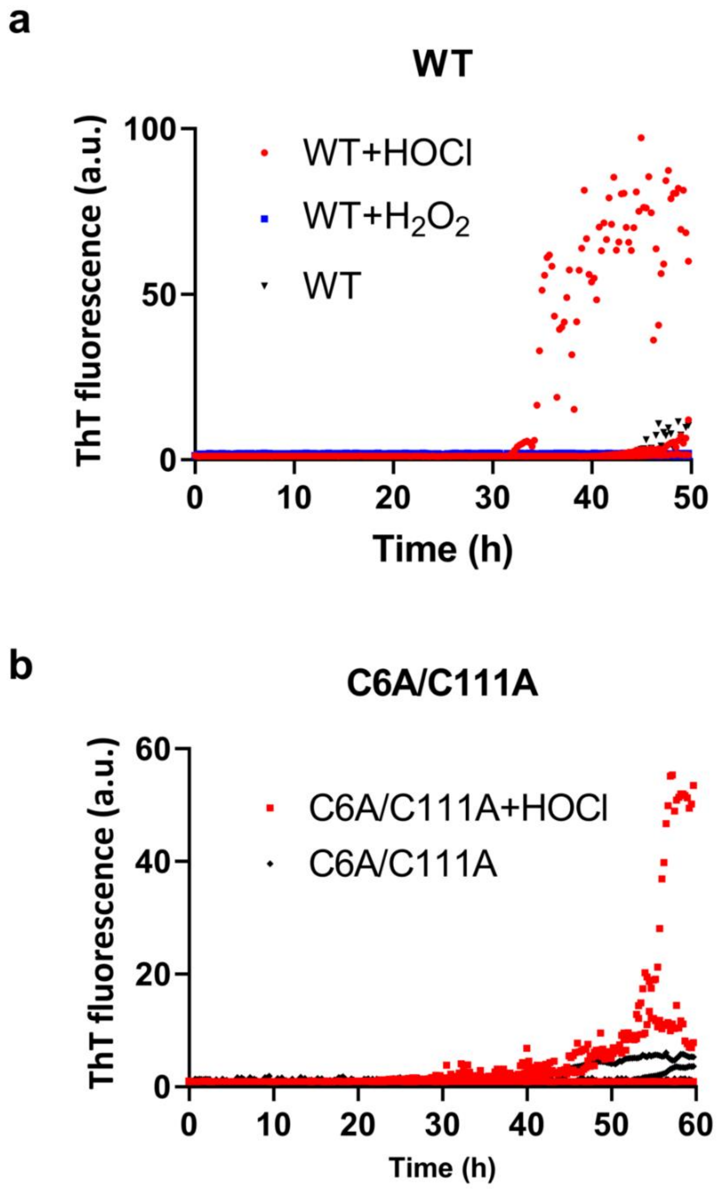

Supplementary Figure 10. Amyloid-like filament formation of SOD1 proteins treated with HOCl or H_2O_2.

ThT fluorescence intensities represent the filament formation of wild-type SOD1 protein (**a**) or C6A/C111A mutant SOD1 protein (**b**). Each protein was treated with HOCl (red), H_2O_2 (blue), or without any ROS species (black). Individual raw datasets of the three independent experiments are presented in the same color.

a

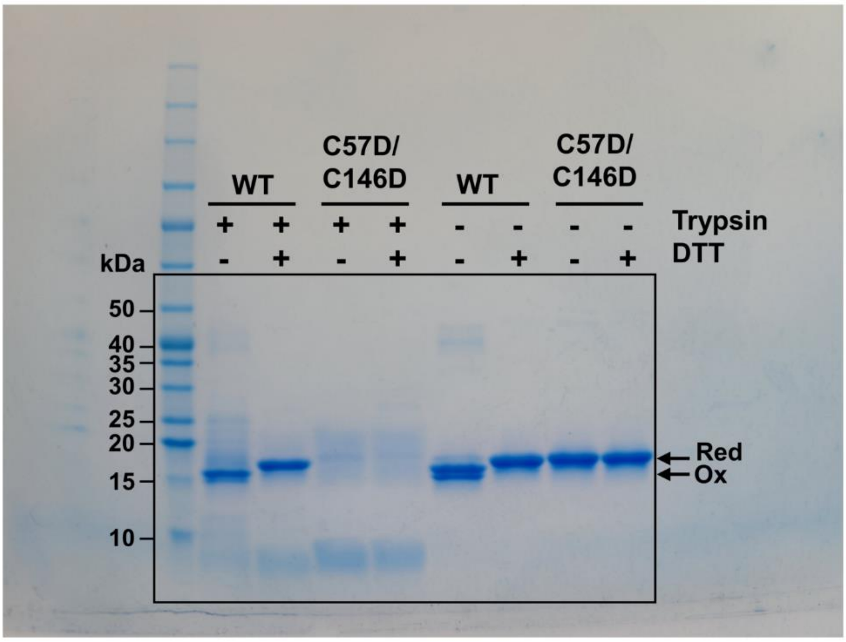

b

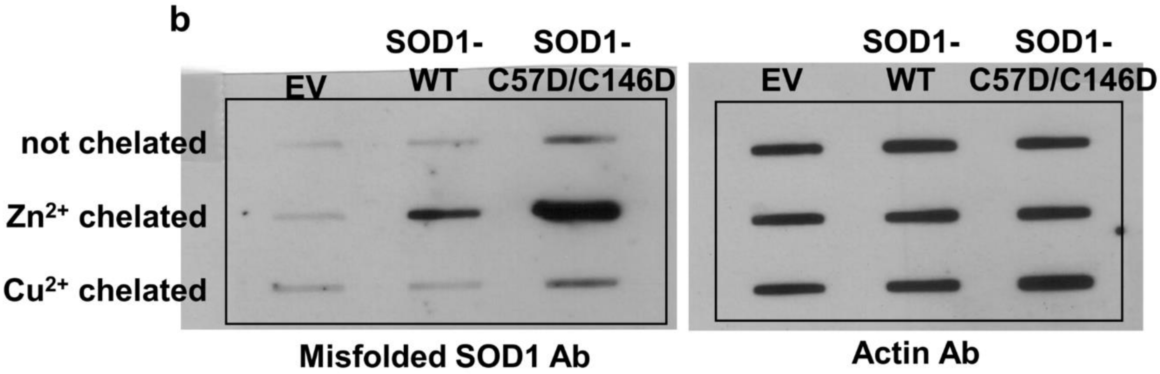

Supplementary Figure 11. The uncropped and unedited blot/gel images.

a. Uncropped image of representative SDS-PAGE of SOD1 proteins digested with trypsin as shown in Fig. 1e. Boxed area represents the cropped part of the SDS-PAGE image.

d. Uncropped images of representative dot blots of misfolded SOD1 expressed in SK-N-SH cells and detected by anti-misfolded SOD1 antibody (left) or anti-actin antibody (right) as shown in Fig. 2a. Boxed areas represent the cropped part of the dot blots.

Supplementary References

1 Rasband, W. S. (Bethesda, MD, 1997).

2 Baek, Y. *et al.* Structure and function of the hypochlorous acid–induced flavoprotein RclA from Escherichia coli. *Journal of Biological Chemistry* **295**, 3202-3212 (2020).
